# Molecular and biotechnological characteristics of proteolytic activity from *Streptococcus thermophilus* as a proteolytic lactic acid bacteria to enhance protein-derived bioactive peptides

**DOI:** 10.3934/microbiol.2023031

**Published:** 2023-08-07

**Authors:** Srisan Phupaboon, Farah J. Hashim, Parichat Phumkhachorn, Pongsak Rattanachaikunsopon

**Affiliations:** 1 Tropical Feed Resources Research and Development Center (TROFREC), Department of Animal Science, Faculty of Agriculture, Khon Kaen University, Khon Kaen 40002, Thailand; 2 Department of chemistry, College of Science, University of Baghdad, Baghdad 10071, Iraq; 3 Department of Biological Science, Ubon Ratchathani University, Warin Chamrap, Ubon Ratchathani 34190, Thailand

**Keywords:** *Streptococcus thermophilus*, bioactive peptides, fish hydrolysate, PLAB starter, antimicrobial activity

## Abstract

The demand for healthy food items with a high nutrient value of bioavailability and bioaccessibility has created a need for continuous development of technology and food ingredients like bioactive peptides. This study aimed to investigate seven proteolytic lactic acid bacteria (PLABs) isolated from the *plaa-som* (fermented fish) sample originated from silver BARB species for production of proteolytic enzymes. Proteolytic enzymes produced by (PLABs) were used further to create potent bioactive peptides by hydrolyzing proteins throughout PLAB-probiotics enhancer. Protein derived-bioactive peptides was tested the proteolytic activity on different protein sources and examined bioactivities including antioxidative and antimicrobial effect for further use in functional foods. Results of screened-PLAB strains showed high proteolytic activity namely *Streptococcus thermophilus* strains (KKUPA22 and KKUPK13). These strains have proteolytic system consisting of extracellular and cell-bound enzymes that used for degrading protein in fish flesh protein (FFP) and skim milk (SKM) broth media. Proteolytic activity of tested bacterial enzymes was estimated after incubation at 45, 37, and 50 °C. Furthermore, FFP hydrolysates were formed with various peptides and has small molecular weights (checked by SDS-PAGE) in the range of10.5 to 22 kDa), exhibiting strong activity. Data revealed that *S. thermophilus* strains (KKUPA22 and KKUPK13) had high antioxidant activity in term of 2,2-diphenyl-1-picrylhydrazyl (DPPH), 2,2-azinobis-(3-ethylbenzothiazoline-6-sulfonate) (ABTS) radical-scavenging inhibition, and ferric reducing antioxidant power (FRAP) reducing power capacity. Both strains (KKUPA22 and KKUPK13) of *S. thermophilus* have higher antimicrobial activity against Gram-negative bacteria than against Gram-positive bacteria. We have confirmed presence of proteolytic (*prt*) gene regions in *S. thermophilus* strains using specific primers via PCR amplification. Results showed highest homology (100%) with the *prt*S gene of *S. thermophillus* located on the cell envelope proteolytic enzymes (CEPEs) such as serine proteinase. Therefore, it concluded that the proteolytic system of tested PLAB strains able to generate bioactive peptides-derived proteins having active biological property, good mechanism of degradability, and bioaccessibility for further use in catalyzing protein of functional foods.

## 1. Introduction

Proteolytic lactic acid bacteria (PLAB) are spontaneously fermentative bacteria containing numerous groups of lactic acid bacteria (LAB) which presented in various fermented food sources. PLAB were able to produce protein hydrolysates based on their proteolytic activity and/or bioactive peptide-derived proteins. However, during the production of such bioactive proteins; proteinase, and peptidase enzymes were involved [1]. Traditionally, LAB characteristics are commonly defined as Gram-positive, catalase-negative, nonsporulating, aerotolerant, acid-tolerant, nutritionally fastidious, strictly fermentative organisms that lack cytochromes and produce lactic acid as the main end-product of carbohydrate metabolism. The genera are typically contained *Aerococcus, Carnobacterium, Enterococcus, Lactobacillus, Lactococcus, Leuconostoc, Oenococcus, Pediococcus, Streptococcus, Tetragenococcus, Vagococcus*, and *Weissella* [2]. Additionally, LAB and yeast are generally recognized as safe (GRAS) for both human and animals with several positive effects on human health, their significance is constantly expanding [3],[4]. There are also displayed a multi-functional effect in functional foods such as probiotic properties, possess antioxidative and/or antimicrobial activities, increasing host immune system, and synthesis the nutrients or nutraceuticals through protein digestion [3]–[5].

Bioactive peptide-derived proteins have major impacts on the functionalization of protein meals to enhance health benefits through metabolism proteolysis of PLABs, especially *Streptococcus thermophilus*. Different strategies were followed in order to modify the peptide-derived proteins' organoleptic qualities such as: (i) increases the amount of vitamins, amino acids, and polysaccharides; (ii) reduces the immunoreactivity of proteins by hydrolyzing allergenic epitopes; and (iii) adds additional advantages like health-promoting molecules through the production of bioactive peptides including antihypertensive, immunomodulatory, hypocholesterolemia, antioxidative, and antimicrobial [6]–[8].

Metabolism of protein via proteolysis step leads to produce bioactive peptides with 2–20 amino acids. This proteolysis can be done by proteolytic system of PLABs. Those active peptides transported to be cell envelope proteolytic enzymes (CEPEs) via an oligopeptide transport system [9]. Several studies have been reported proteolytic system as greatest-recognized system of PLABs throughout molecular, biological and mechanism properties [1],[9]. The proteolytic enzymes involved in their systems are divided to: (i) an extracellular enzyme and/or CEPEs; (ii) peptide transport systems; and (iii) intracellular peptidases [1],[9],[10]. Numerous research findings of CEPE-encoding DNA gene expression found that in various of PLABs based on muscular protein or casein hydrolysis, consisting of *prtS* identified in *S. thermophilus* [7],[11], *prtP* originated from *Lactococcus lactis* subsp. *cremoris, Lactiplantibacillus plantarum*, and *Lacticaseibacillus paracasei* ssp. *paracasei* [12],[13], along with *prtH* and *prtR* obtained from *Lactibacillus helveticus* and *Lactobacillus rhamnosus* [14].

Therefore, current study aimed to investigate molecular and biotechnological characteristics of PLABs isolated from the *plaa-som* (fermented fish) for production of proteolytic enzymes.In details, the proteolytic activity of *S. thermophilus* strains (KKUPA22, KKUPA80, KKUPK13, KKUPJ63, KKUPJ65, KKUPL85, and KKUPL90) isolated from *plaa-som* products was tested. Proteolytic enzyme activity of the PLABs was estimated based on characterization of enzymes locations inside the cell under stress conditions, protein degradability was investigated by SDS-PAGE electrophoresis, and analyzed activity of produced hydrolysates effect as antioxidant and antimicrobial activities. Besides, amplification and identification of the *prt* gene encoding-CEPE in PLAB were considered using specific primers via PCR technique.

## 2. Materials and methods

### 2.1. Microorganisms and culture condition

Briefly describe on bacterial strains, particularly lactic acid bacteria (LAB) were isolated from different *plaa-som* samples (fermented fish: n = 13 samples; 112 isolates) of silver BARB fish species and collected from local market around Khon Kaen province in Thailand, which supported by the previous research of Phupaboon et al. [15],[16]. Seven candidates of LAB, *Streptococcus thermophilus* namely KKUPA22, KKUPA80, KKUPK13, KKUPJ63, KKUPJ65, KKUPL85, and KKUPL90 used in this study. All strains were identified using 16S rDNA sequencing via universal pair-primers, 27-F (AGAGTTTGATCMTGGCTCAG) and 1389-R (ACGGGCGGTGTGTACAAG). Homologous relationships of biological sequences showed a high similarity index of 98–99% with *Streptococcus thermophilus* at different accession no. consisting of MG589389, MF784246, MF784197, MF784196, MF784109, MF784143, and MF784144, after aligned the sequence with NCBT database. Additionally, three reference strains of LAB used in this study (*Lactococcus lactis* TISTR 1464, *Lactiplantibacillus plantarum* TISTR 854, and *Lacticaseibacillus casei* TISTR 1463) were obtained from Thailand Institute of Scientific and Technological Research (TISTR), Pathum Thani, Thailand. Additionally, three pathogenic bacteria including *Escherichia coli* TISTR 073, *Staphylococcus aureus* TISTR 029, and *Enterobacter aerogenes* TISTR 1540 cultured in nutrient broth overnight to use in antimicrobial activity test for checking some characteristics of the probiotic property. Pure cultures of LAB strains were activated on modified lactose-MRS broth [17] and re-subcultured into MRS broth containing 10% (w/v) of sterile skim milk broth (HiMedia, Maharashtra, India) and incubated at 37 °C for 24 h. After activation, two successive transfers into the same medium were performed. During this process, Gram's staining, catalase test, milk curd formation, and viable counts were performed and evaluated according to the method described by Phupaboon et al. [15] and Cao et al. [19].

### 2.2. Primary screening of proteolytic lactic acid bacteria

The primary screening of proteolytic lactic acid bacteria (PLAB) was slightly modified from the previous study of Phupaboon et al. [15]. Each strain of LABs and reference strains was screened on a modified-MRS agar plate containing 10% of sterile skim milk (SKM) and flesh fish protein (FFP) powder broth via sterilization method by autoclaved at 110 °C for 15 min [18] for observing the proteolytic activity or the formation of halo zones around the colony using spot techniques on those modified media plates. The plates were incubated at 37 °C for 48 h. The LAB colonies were grown on an agar plate and the proteolytic activity was measured by calipers and expressed as millimeters (mm).

### 2.3. Proteolytic activity and protein hydrolysate produced from PLABs

The investigation of proteolytic activity of PLAB strains at different cellular localization was a slightly modified method described by Cao et al. [19] and Keay et al. [20]. All prospective PLAB and three reference strains were cultured in 10% of SKM broth (SKMB) and FFP broth (FFPB) without MRS medium in condition and incubated at 37 °C for 24 h designed for further use to determine the proteolytic activity and bioactive compound from their crude protein hydrolysate samples. One milliliter of culture broth was filtered and centrifuged at 10,000 rpm at 4 °C for 2 min to separate the cell pellet and supernatant. And then each portion was used to determine the proteolytic activities: extracellular and cell-bound enzymes.

Proteolytic activity was analyzed according to the method described by Genay et al. [14] with slightly modification by applying different temperature conditions: 37, 45, and 50 °C for 30 min through the enzymatic reaction. The first portion of the supernatant was determined for extracellular (free cell) activity. Then, the second portion of the cell pellet was washed twice and re-suspended with 1 mL of 100 mM PBS buffer then assessed for cell-bound (cell-surface-bound extracellular) activity. One unit of proteolytic activity was defined as the amount of enzyme required to produce 1.0 µmole of tyrosine from casein in one minute under the defined assay conditions. Proteolytic activity unit was expressed as U/mL.

### 2.4. Tolerance in stimulated gastrointestinal tract conditions

The experiment was performed based on previous described method of Phupaboon et al. [17] and Cheon et al. [21] to investigates the existed strains in stimulated stomach conditions tolerance. Two potential *S. thermophilus* strains KUUPA22 and KKUPK13 were incubated overnight in MRS broth at 37 °C. Selected PLAB strains of *S. thermophilus* KKUPA22 and KKUPK13 strains are potent PLAB bacteria. Therefore, they were selected to study the properties in terms of being a safe microorganism, such as testing for probiotic bacteria and being able to function as bacteria. For the initial cell suspension of each strain (approximately 6 log CFU/mL) was added to modified MRS broth together with synthetic gastric juice at the different conditions of 0.3% lysozyme from egg white (Sigma-Aldrich, Missouri, USA), adjusted to pH 3.0, then added 0.3% OX-bile salts (HiMedia, Maharashtra, India) and incubated at 37 °C for 24 h, respectively. After each treatment, the viable cells (Log CFU/mL) were counted by optimal dilution factor and plating on MRS agar using the drop plate technique. Each treatment was performed in triplicate and the survival rate (%) was calculated according to the following equation (1):

$$\text{Survival rate}\left(\%\right)=\left[\text{Log CFU}/{\text{mL}}_{\left(\text{treatment}\right)}/\text{Log CFU}/{\text{mL}}_{\left(\text{initial}\right)}\right]\times \text{1}00$$ (1)

### 2.5. Gel electrophoretic analysis of protein hydrolysate

Investigate the alteration of bioactive protein hydrolysates was achieved according to the method of Phupaboon et al. [17],[18]. The resulted peptide derived from protein hydrolysates and protein ladder (Chromatein prestained protein ladder, Vivantis, Malaysia) were submitted to sodium dodecyl sulfate-polyacrylamide gel electrophoresis (SDS-PAGE) using a Mini-Protean II unit (Bio-Rad, CA, USA) consistent with the standard method of Laemmli [22]. The amount of acrylamide in the stacking and resolving gels was employed at 5 and 15%, respectively. After the separation, the SDS-polyacrylamide obtained gel was stained with 0.1% Coomassie^®^ brilliant blue R250 and de-stained in an overnight procedure using a moderate shaker speed and a 10:40% combination of acetic acid and methanol in water. The band area was measured through the gel documentation system (Bio-Rad, CA, USA).

### 2.6. Bioactive protein hydrolysate extraction

The FFP and SKM hydrolysates (FFPHs and SKMHs) were extracted using microwave-assisted extraction following method described by Phupaboon et al. [18] with some modification. The crude protein samples (5 g) which obtained from hydrolysis step were extracted under microwave equipment in the optimal condition of 100 W, maximum temperature ≥ 60 °C for 20 min and homogenized using a homogenizer IKA T25 digital ultra turrax (MiliporeSigma, Darmstadt, Germany) with 25 mL of 100 mM phosphate buffer pH 7.0 in an ice bath at 4 °C for 30 min. After that, each homogenate was separately centrifuged at 10,000 rpm at 4 °C for 5 min and lyophilized by freeze-drying technique for bioactive protein hydrolysate powders [19].

### 2.7. Antioxidant activity of bioactive protein hydrolysate

The approach described in the protocol of Phupaboon et al. [16],[18] was modified by three methods, including DPPH, ABTS radical-scavenging activity [23]–[25] and FRAP reducing power capacity [26] were used to measure the antioxidant activity from bioactive protein hydrolysates. All methods were run through a PerkinElmer microplate reader (PerkinElmer, Massachusetts, Germany). Each lyophilized FFPH and SKMH powder was dissolved in a methanol solution at a concentration of about 10 mg/mL, and its antioxidative capacity was measured at 517, 734, and 595 nm, respectively. All analyses were carried out in triplicate and calculated in percentage of DPPH and/or ABTS radical-scavenging inhibition. Obtained results compared with vitamin C (ascorbic acid) as a positive control and expressed in mmol Trolox equivalent (TROE/g) samples in term of FRAP reducing power capacity.

### 2.8. Antimicrobial activity of bioactive protein hydrolysate

The swab paper disc method was modified to assess each LAB's antimicrobial activity against *E. coli* TISTR 073, *S. aureus* TISTR 029, and *Ent. aerogenes* TISTR 1540 according to the method of Phupaboon et al. [17] and Alsaraf et al. [27].

About 0.2 mL of the cultured-pathogenic strain (10^5^ CFU/mL) which obtained after incubation in nutrient broth (HiMedia, Maharashtra, India) for 18 h was spread with a sterile swab on nutrient agar, and then sterile filter paper discs (6 mm, Whatman, Maidstone, UK) in diameter were placed on the agar. Each of sterile bioactive protein hydrolysate in the supernatant (40 µL) was spotted on the paper disc. Streptomycin disc (15 µg) was used as a positive control. After incubation, the diameter of the inhibition zone around the disc was measured by calipers and expressed as millimeters (mm) obtained from triplicates.

### 2.9. Amplification of proteolytic (prt) genes of PLAB strains

After incubation step for 16 h; the genomic DNA (gDNA) was extracted from the cultured colony of each LABs in the stationary phase. The GF-1 Kit general protocol (Vivantis, Malaysia) was used in extraction process. The total gDNA obtained was suspended in 50 µL of Elution buffer and stored at −20 °C till further use in DNA amplification. Specific primer sequences were designed from the cell-envelope proteolytic gene; this primer which consisting of *S. thermophilus* (accession no. ADB77872), *L. paracasei* (accession no. M83946), and *L. lactis* subsp. *cremoris* (accession no. CAA01252) was obtained from the NCBI microbial genome database. Primer sequences with approximately 388 bp amplicons were degenerated by Novogene, Singapore to amplify the conserved targeted region surrounding the active site of the proteolytic gene that consisted of *prt1-F*: 5′-AACTGTGGTCGCTCCTCGT-3′ and *prt2-R*: 5′-TGCTAACGGGACAGGTGAC-3′. PCR was achieved according to the procedure used by Lozo et al. [12] and Phupaboon et al. [15] the final volume of reaction was 50 µL and contain 4 µL of gDNA template, 2.5 µL (10 µmol/µL) of each primer, 25 µL of 2X Taq Master Mix, and 16 µL of deionized water. In this study, *Enterococcus faecium* TISTR 2058, a non-proteolytic lactic acid bacterium, was used as a negative control to confirm the high specificity of the primer pair to the *prt* gene. Amplification of *prt* gene was accomplished according to the following condition: 5 min of initial denaturation at 95 °C, 30 cycles of three steps of 1 min (denaturation step at 95 °C, annealing step at 58 °C, extension step at 72 °C), and final extension step at 72 °C for 10 min. The PCR product was analyzed by 1% (w/v) agarose gel electrophoresis and visualized using a UV transilluminator (Bio-Rad, CA, USA). All attained PCR fragments were purified according to the QIAquick PCR purification Kit (Qiagen, Hilden, Germany) and sequenced by the Macrogen (Macrogen, Seoul, South Korea). The sequence was analyzed in the NCBI database using BLAST programs.

### 2.10. Statistical analysis

All tests were achieved in this study in triplicate. Data were expressed as mean ± SD values. Analysis of variant (ANOVA) was used to evaluate the differences among the treatments by Duncan's multiple range tests (DMRTs) with p value < 0.05 using SPSS-KKU Statistics v.27.

## 3. Results and discussion

### 3.1. Screening of LAB-producing proteolytic activity

**Figure 1.**
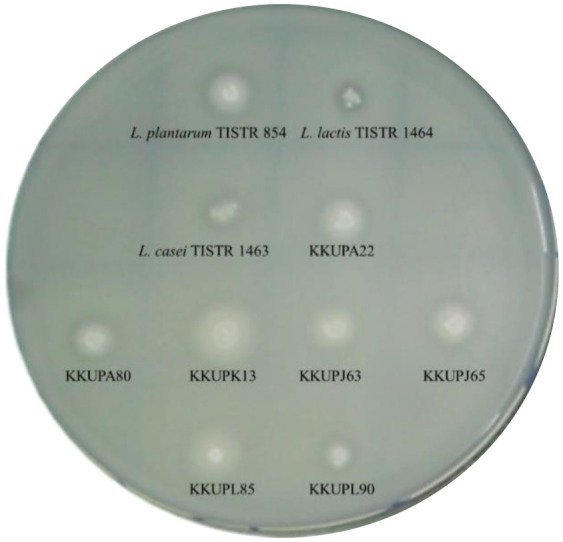
A display of a halo zone surrounding colonies of each pure PLAB strain after growing on modified MRS-skim milk agar (MRS-SKMA).

Table 1 shows Gram-positive, coccal sharp, and catalase-negative of seven homo-fermentative LAB, namely *S. thermophilus* strains (KKUPA22, KKUPA80, KKUPK13, KKUPJ63, KKUPJ65, KKUPL85, and KKUPL90) obtained from *plaa-som* (fermented fish) products along with three reference strains (*L. lactis* TISTR 1464, *L. plantarum* TISTR 854, and *L. casei* TISTR 1463). All strains were primarily screened for high proteolytic activity using a modified-MRS agar plate which contain 10% of fish flesh protein (FFP) and skim milk (SKM) media (Figure 1). For the typical phenotypic characteristics of PLABs, different sizes of halo zones resulting from protein hydrolysis were observed around colonies of the bacteria. [1],[5],[10],[19]. The current results reported that each LAB-producing proteolytic enzyme, called PLAB strain, had halo zones in a different media including FFP and SKM agar. The PLABs had different sizes of extracellular proteolytic activity (EPA)-producing halo zone in the range of 9 to 12 mm in size, respectively. The FFP medium agar that adopted for detecting extracellular proteolytic activity (EPA); showed more degradation compared with SKM agar. A bright halo zones surrounding the colonies were used to determination (EPA) as shown in Table 1. In case of LAB strains; the hydrolyzing effect on myofibrillar and/or sarcoplasmic proteins obtained from fish flesh more potent than its effect on proteins of constitutive skim milk. Similarly, Cao et al. [19] and Drosinos et al. [28] screened 64 LABs isolated from fermented sausages for their proteolytic activity and found several LABs isolates having proteolytic activity namely *L. plantarum*, *L. curvatus*, and *L. casei*. The results showed strong proteolytic activity to hydrolyze sarcoplasmic and myofibrilla proteins in ranges of 8.27–28.51 and 9.03–28.40 mm, respectively. Several studies have reported the large halo zones of *L. bulgaricus* and *S. thermophilus* producing EPA, between 4.5 and 4.6 mm on the SKMA medium [29]. These differences can be explained in term of proteins composition; sarcoplasmic and myofibrillar protein originates from native traditional sources (meat, pork, and fish) and also depended on the ability of proteolytic enzyme to degrade protein by the action of PLAB-producing proteolytic activity [19],[30],[31]. Notably, proteolysis process produces free amino acids and small peptides which are particularly crucial for rapid microbial growth and acidification during fermentation. Besides, those amino acids and peptides well-known as active molecules used as a precursor for the flavor development of leavened baked goods that raises during the fermentation process [32]. Many previously studies have screened proteolytic enzymes-producing strains by using different protein sources as substrate including constitutive skim milk, soybean, wheat flour protein fractions, and raw fish-juice media. Those kinds of proteins used to test the activity of LAB-producing proteolytic enzymes in the natural protein sources [33]–[35].

**Table 1.** Morphological, biochemical characteristics and proteolytic activity of *S. thermophilus* strains and three reference strains determined by the halo zone surrounding the colony on different protein source agar medium.

LAB strains	Cell shapes	Gram stains	Catalase test	Curd formation	Proteolytic activity (mm)	
					MRS-FFPA	MRS-SKMA
KKUPA22	cocci	+	-	+	+3	+1
KKUPA80	cocci	+	-	+	+3	+1
KKUPK13	cocci	+	-	+	+3	+1
KKUPJ63	cocci	+	-	+	+3	+1
KKUPJ65	cocci	+	-	+	+3	+1
KKUPL85	cocci	+	-	+	+3	+1
KKUPL90	cocci	+	-	+	+3	+1
*L. lactis*	cocci	+	-	+	+3	+3
*L. plantarum*	rod	+	-	+	+3	+3
*L. casei*	rod	+	-	+	+3	+3

Note: (+) as positive test, (-) as negative test, (+1) indicated the diameter size of the halo zone between 9.0 to 10.0 mm, (+2) indicated the diameter size of the halo zone between 10.1 to 11.0 mm, and (+3) indicated the diameter size of the halo zone between 11.1 to 12.0 mm.

### 3.2. Proteolytic activity of PLAB strains

**Figure 2.**
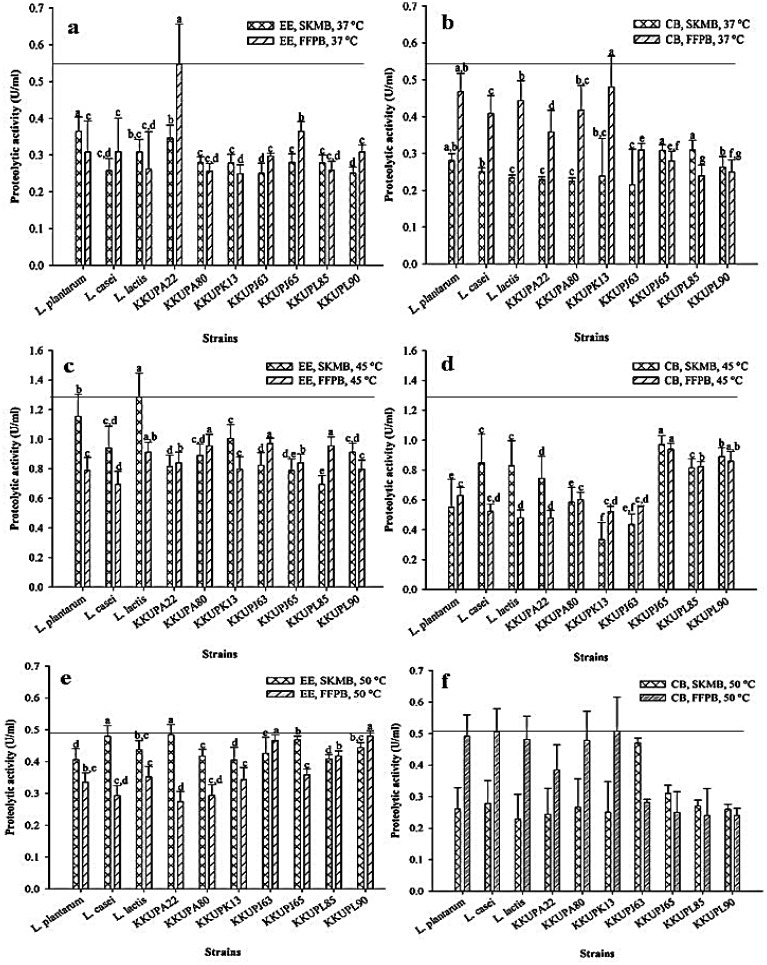
Kinetics production and localization of proteolytic activity from each selected PLABs and reference strains after being cultivated under different conditions of SKMB and FFPB media and thermal enzymatic reaction; (A, B) as determined at 37 °C; (C, D) as determined at 45 °C; (E, F) as determined at 50 °C; EE, extracellular enzyme; CB, cell-bound; SKMB, skim milk broth; FFPB, fish flesh protein broth.

Seven strains of PLAB and three reference strains with high proteolytic activity on modified-MRS containing FFPA agar are shown in Table 1. Under different conditions as shown in Figure 2 (a–f), their produced proteolytic enzymes were found in 2 different locations; extracellular and cell-bound enzymes. Proteolytic activity of Total crude enzymes of each strain was tested including the extracellular (EE) and cell-bound (CB) enzymes obtained from SKMB and FFPB media at different optimal temperature conditions (37, 45, and 50 °C). These data also accord with our earlier observations, which revealed that *Lactobacillus bulgaricus* NCDO 1489 produce proteinase enzyme via chelating substances which showed best activity at pH 5.2–5.8 and 37–50 °C. The enzyme was mostly related to the CB enzymes group, but it could be released from cells in some cases such as during encouraging autolysis or by giving the cells lysozyme therapy. It's associated with the organism's development in milk and had an effective contribution to the production of fermented milk products [36]. The result of *S. thermophilus* KKUPA22 after being cultured in FFPB showed the highest EE activity as at 0.55 U/mL with significant difference at (*p* < 0.05) compared to the selected reference strains results, (0.40–0.48 U/mL) at 37 °C for thermal reaction. Correspondingly, *S. thermophilus* KKUPK13 produced the highest CB activities which was recorded at approximately 0.48 U/mL under a similar condition of proteolytic activity determination as shown in (Figure 2a and b), respectively. Additionally, *S. thermophilus* KKUPK13 after cultured in SKMB showed the highest EE activity at 1.0 U/mL, while *S. thermophilus* KKUPJ65 showed the highest CB activity at 0.9 U/ mL at 45 °C for thermal reaction obtained from both media (Figure 2c–d). Figure 2e and f accomplished that the seven PLAB strains significantly exhibited the EE activity ranging 0.40–0.49 U/mL when cultured in SKMB at 50 °C. Further analysis of the proteolytic activity at 50 °C showed that the *S. thermophilus* KKUPA13 and KKUPA80 was significantly achieved the highest CB activity after cultivation in FFPB at 0.50 and 0.48 U/mL (Figure 2e, f), respectively. Previous study was reported that *L. bulgaricus* produced only CB proteolytic activity during growth on milk medium at 45 °C. optimum proteolytic activity was at 45–50 °C and pH 5.2–5.8. Also, it was inhibited by chelating agents [37]. Similarly, Pailin et al. [29], stated that *L. bulgaricus* could produce higher extracellular cell-bound proteolytic activity than *S. thermophiles* at 37 °C in yogurt culture medium. The ability to produce high yield of proteolytic enzymes among PLAB and various *Bacillus* strains, such as *Bacillus horikoshii*, *Bacillus subtilis*, and *Bacillus licheniformis*, would be suitable for food industrial application [37]. Subsequently, the extensive studies have been performed on serine alkaline protease production, that is significantly more active and stable at considerably higher pH and temperature [38]. Extracellular enzymes and cell-bound activities of PLAB had more interesting than intracellular enzyme. Those enzymes were produced by PLAB to hydrolyze proteins and making smaller peptides, and/or free amino acids for transportation inside the cell to use as a nitrogen source for bacteria growth [10]. Chabasri et al. [39] demonstrated that the LAB strains isolated from available *plaa-som* samples have potentially to be producing-amylolytic lactic acid bacteria (ALAB) and inulin lytic lactic acid bacteria (ILAB). These strains were exhibited two types of both amylolytic and inulin lytic through the EE and CB enzyme activities on modified MRS broth mixed with soluble starch and either garlic or inulin powders. Additionally, these potential strains wereused for developing pure culture starter strains to be used in the food or chemical industries to produce proteolytic, amylolytic, and inulin lytic enzymes [15],[16].

Interestingly, the results obtained from the preliminary analysis of the proteolytic activity of both potent *S. thermophilus* strains (KKUPA22 and KKUPK13) based on FFP hydrolysis at 37 °C demonstrated the suitable conditions for production of the highest EE and CB enzymes activities. The obtained data from the biochemical and physicochemical properties of these PLABs regarding to proteolytic enzymes production are therefore suitable to be studied in terms of different properties related to biotechnology applications including tolerance in the stimulated gastrointestinal tract condition, bioactive activity of protein hydrolysate, protein alteration, and molecular biological of proteolytic genes expression.

### 3.3. Probiotic property of potential PLAB strains

The survival rate of *S. thermophilus* KKUPA22 and KKUPK13 showed no significant differences (*p* < 0.05) and also no effect on the growth resistance in term of the high lysozyme content with the low pH, and the high bile salt concentration as shown in Table 2. The survival rate of these strains was reported in the ranges of 98.7 to 98.2, 89.1 to 88.9, and 100.6 to 100.2%, respectively. These findings are consistent with the outcomes reported by [40] who found that ten *S. thermophilus* strains (st1 to st10) isolated from pickles in China was demonstrated the highest lysozyme resistance by St10 (> 80% survival) while St5 had the highest survival rate (35%), followed by St6 (30%), for tolerance to simulated gastric juice. In terms of bacterial adherence to the Caco-2 cells and resistance to bile salts, St6 was achieved the best effects. Similarly, *S. thermophilus* STI-06 was adopted to determine the effects of carao pulp powder on the bacterial viability, acid tolerance, bile tolerance, and protease activity [41]. According to research on different strains, the probiotic potential of *Lactobacillus* strains is consisting of *Lacticaseibacillus casei*, *Lactiplantibacillus plantarum*, *Lactobacillus acidophilus, Lactobacillus rhamnosus, Lactobacillus delbrueckii*, and *Lactobacillus buchneri* which subjected to evaluation their probiotic properties. Probiotic property results including tolerance to enzymatic digestion, gastric acid, and bile salt conditions were recorded in the ranges of 76.6–100.7, 65.2–100.4, and 60.2–102.4%, respectively [17],[21]. These data of KKUPA22 and KKUPK13 strains represented highly potential and survival rates compared to reference strains. These findings indicate that the functional properties of PLABs are related to increased protein digestibility in the gastrointestinal tract and increase the survival rate of microbial populations in their state as a defense against pathogens by proteins and peptides derived from milk, egg, meat, and fish [16]–[18],[42].

**Table 2.** Survival rate of different *S. thermophilus* and referent strains under tolerance stimulated gastrointestinal tract conditions.

PLAB strains	Survival rate (%)		
	Lysozyme (0.3%)	pH (3.0)	Bile salt (0.3%)
KKUPA22	98.7 ± 2.1^a^	89.1 ± 1.1^a^	100.6 ± 1.2^a^
KKUPK13	98.2 ± 1.2^a^	88.9 ± 1.0^a^	100.2 ± 1.0^a^
*L. lactis*	84.6 ± 1.2^c^	78.4 ± 1.0^b^	89.3 ± 2.1^b^
*L. plantarum*	92.0 ± 3.3^b^	66.3 ± 1.2^c^	100.1 ± 2.1^a^
*L. casei*	90.4 ± 4.4^b,c^	66.8 ± 2.0^c^	100.0 ± 2.2^a^

Values are expressed as the mean ± SD (n = 3); ^a,b,c^ indicated as a superscript letter indicating significant differences in each column (*p* < 0.05 by Duncan's test).

### 3.4. Proteolysis and protein alteration

**Figure 3.**
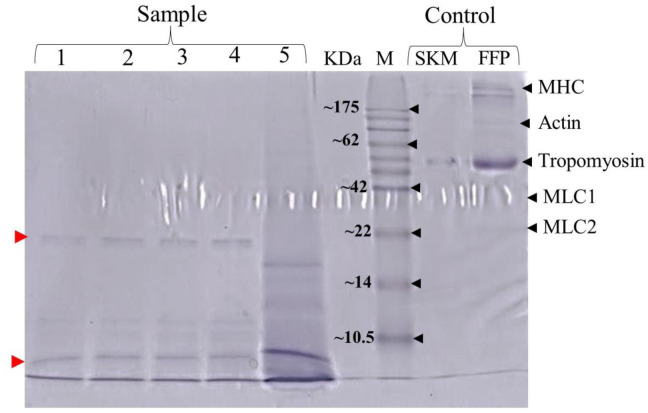
SDS-PAGE profile and protein alteration of FFP hydrolysates after incubated with each potential PLABs: (1), *S. thermophilus* KKUPA22; (2), *S. thermophilus* KKUPK13; (3), *L. lactis* TISTR 1464; (4), *L. casei* TISTR 854; (5), *L. plantarum* TISTR 1463; M, protein ladder (kDa); SKM, skim milk protein used as a negative control; FFP, fish flesh protein used as a negative control; MHC, myosin heavy chain; MLC1 and MLC2, myosin light chain 1 and 2.

For the Figure 3 shows SDS-PAGE profile of FFP hydrolysates after incubated with PLABs of *S. thermophilus* strains KKUPA22 (Lane 1) and KKUPK13 (Lane 2) compared with three referent strains of *L. lactis* TISTR 1464 (Lane 3), *L. casei* TISTR 85 (Lane 4), and *L. plantarum* TISTR 1463 (Lane 5). This finding is in agreement with Phupaboon et al. [16],[18] and Kasankala et al. [43] mentioned previous studies found that fish hydrolysates of silver barb and/or silver carp fish species were generated from small proteins or peptides throughout fermentation based on proteolysis activity that created via proteolytic enzyme-producing LAB. The fish hydrolysates were resulted from the degradation of sarcoplasmic and myofibrillar proteins with high molecular weights consisting of myosin heavy chain, actin, tropomyosin, and different sizes of myosin light chains. The resulting short peptide content reached 225.5 to 488.6 µmole tyrosine/g sample. For the current results as shoen in Figure 3, the muscular proteins ranging from 22 to 175 kDa were degraded into different by-product molecular weight proteins ranging from 10.5 to 22 kDa. In Figure 3, small proteins, or peptides smaller than 10 kDa were observed during the degradation of proteins by the proteinase produced by PLAB strains particular *S. thermophilus*. The present findings are consistent with several previous reports. Phupaboon et al. [17] found that pork protein hydrolysates generated from sausage fermentation activated by different genera of *Lactobacillus* sp. consisted of peptides with molecular weights ranging from 1.2–22 kDa. These peptides displayed an important role in functional food and bioacccessibility in term of probiotic or prebiotic potential, antioxidative and antimicrobial activities [17],[19],[21],[42],[44]. Similarly, Phupaboon et al. [16],[18] and Binsan et al. [24] found small peptides smaller than 10 kDa during the production of protein hydrolysates from traditional fermented fish (plaa-som) products. The fish/marine-derived small peptides (less than 10 kDa) were also reported to have bioactive activity in ternm of antioxidant and antimicrobial peptides [44],[45].

### 3.5. Antioxidant activity of FFP hydrolysate

According to above SDS-PAGE profile of FFP hydrolysates after incubation with different *S. thermophilus* (KKUPA22 and KKUPK13) and three referent strains, the hydrolysates would have potential to be bioactive peptides such as an antioxidant and/or antimicrobial peptides. These outcomes support the idea of fish peptides effectiveness mentioned peptides have been degraded by enzymes and display a variety of physicochemical characteristics and biological functions associated with their molecular weight and amino acid composition. Therefore, one of the most crucial aspects in creating bioactive peptides with the appropriate biological activities is the molecular weight of the peptide [42],[45],[46]. The results, as shown in Table 3, indicate that the antioxidant capacity of *S. thermophilus* KKUPA22 showed the highest DPPH and/or ABTS radical-scavenging inhibition and FRAP reducing power capacity with similar effect to *S. thermophilus* KKUPK13 ranged at 80.2–79.9%, 82.2–81.6%, and 74.6–68.9 mmole TROE/g sample, respectively. While all the referent strains exhibited the highest FRAP reducing power capacity (55.6 mmole TROE/g sample) and more than both tests (DPPH and ABTS radical-scavenging inhibition) in the ranges of 32.7–47.2% (Table 3). The most striking result to emerge from the data is that all PLABs used as a potent probiotic and able to present distinctive antioxidant defenses, such as antioxidant enzymes and antioxidant dietary ingredients, and function to eliminate or replace damaged substances. Recent researches have shown that various fish or meat protein hydrolysates generated from fermentations of different LAB species, *L. plantarum*, *L. casei*, *Latilactobacillus sakei*, *Lactilactobacillus curvatus*, *Pediococcus acidilactici*, *Entercoccus gallinarum*, and *S. thermophilus*, show potential antioxidant activity [17],[47],[48]. Zhang et al. [49] reported that the multiple fractions of antioxidant peptides resulted from jellyfish protein hydrolysates fermented with fermentative-LAB. Therefore, identified peptides including (AAGPAGPDGR, GCGLGDPPGHGK, WGPGPPGDLGAA, and SY) exhibited the highest efficiency to damage different types of free radicals; DPPH^•^, ^•^OH, and O_2_^•^ with IC_50_ values at 84.623 µM, 1177.632 µM, and 456.662 µM, respectively. In another study of Dharmisthaben [50], establish that eleven novel peptides consisting of LLILTC, AVALARPK, YPLR, LSSHPYLEQLYR, TQDK, LAVP, NEPTE, VSSTTEQK, LAVPIN, KP-VAIR, and LLNEK discovered in a *L. plantarum* resulted from fermented camel milk were described to have efficiency in the scavenging activity against ABTS radical, hydroxyl radical, and superoxide radicals. Likewise, two peptides NEDNHPGALGEPV and KVLPVPQQMVPYPRQ from camel milk protein, shown antioxidant activity against DPPH^•^ (IC_50_ 0.04 and 0.02 mg/mL), ^•^OH (IC_50_ 0.05 and 0.05 mg/mL), ABTS^•+^ (IC_50_ 0.1 and 0.01 mg/mL), and O_2_^•-^ (IC_50_ 0.045 and 0.3 mg/mL) [51]. In addition, previous research have found that the antioxidant peptides were exploited their bioactive peptides to reduce the production of reactive oxygen species through activating intestinal absorption and metabolism by endogenous cellular and *in vivo* animal models [52],[53].

### 3.6. Antimicrobial activity of FFP hydrolysate

Antimicrobial results of current study are consistent with those of Reddy et al. [54] and Rajanbabu et al. [55], who stated that the majority of antimicrobial peptides contain less than 50 amino acids, and there have a molecular weight below 10 kDa, which generated by enzymatic hydrolysis. The antimicrobial activity of FFP hydrolysate obtained from each strain was shown in Table 3. *S. thermophilus* strains (KKUPA22, KKUPK13), and three referent strains as well exhibited wide range of activity against Gram-positive and/or -negative of *E. coli*, *S. aureus*, and *Ent. aerogenes*. Mainly, KKUPA22 and KKUPK13 strains displayed the highest diameter of inhibition zone in ranges 10.4–20.2 mm. Moreover, all referent strains exerted significant inhibitory activity against Gram-negative bacteria of *E. coli* and *Ent. aerogenes* than Gram-positive bacteria of *S. aureus* (Table 3). Similarly, three formulations of *Nham* protein hydrolysates: NPH-*nham*1, NPH-*nham*2, and NPH-*nham*3 fermented with different potent probiotic *Lactobacillus* sp. was reported in the antimicrobial spectrum against *E. coli, S. aureus, Ent. aerogenes*, and *Salmonella typhimurium* in average of clear zone at 22.0, 18.0, 17, and 16.0 mm, separately [17]. Several studies described fish antimicrobial peptides against Gram-negative and -positive pathogens due to their antibacterial or bacteriostatic properties [56]. In other study of Chang et al. [57] showed that hepcidin TH1–5, an antimicrobial peptide, synthesized from tilapia, was also exhibited antitumor activity against several tumor cell lines. Additionally, an extracellular neutral protease originates from fungus *Trichoderma harzianumwas* used to hydrolyze fish protein beside further purification of a peptide with a strong antibacterial property, and the sequence was determined to be FPIGMGHGSRPA [58]. Correspondingly, marine-derived fungus named *Trichoderma* sp. JWM29-10-1 was created five new polyketides (1, 2, and 6–8) and seven known compounds (3–5 and 9–12), mainly compound 1 which was significantly inhibits Gram-positive organisms such as methicillin-resistant *Staphylococcus aureus* (MRSA), *Enterococcus faecalis*, and vancomycin-resistant *Enterococcus faecium*, which pose a serious threat to human health [59].

**Table 3.** Antioxidant and antimicrobial activities of FFP hydrolysate after incubated with different *S. thermophilus* and referent strains.

PLABs strains	Antioxidant activity			Antimicrobial activity (mm)		
	DPPH (%)	ABTS (%)	FRAP (mmole/g)	*E. coli*	*S. aureus*	*Ent. aerogenes*
KKUPA22	80.2	82.2	74.6 ± 1.7 ^a^	20.2 ± 2.0^a^	12.0 ± 0.0^a^	19.4 ± 2.0^a^
KKUPK13	79.9	81.6	68.9 ± 1.0 ^b^	19.9 ± 1.0 ^a,b^	10.4 ± 2.0^b^	19.4 ± 2.0^a^
*L. lactis*	41.8	33.2	55.6 ± 3.1^c^	12.2 ± 1.0 ^c^	8.0 ± 0.0^c^	10.6 ± 1.0^c^
*L. plantarum*	47.2	44.5	55.0 ± 1.4 ^b^	14.6 ± 2.0 ^b^	8.2 ± 2.0^c^	12.4 ± 2.0^b^
*L. casei*	43.4	32.7	54.7 ± 2.4 ^b,c^	14.2 ± 0.0 ^b^	8.4 ± 2.0^c^	12.0 ± 0.0^b^

DPPH as a DPPH radical-scavenging inhibition; ABTS as a ABTS radical-scavenging inhibition; FRAP as a ferrous reducing antioxidant power; values are expressed as the mean ± SD (n = 3); ^a,b,c^ indicated as a superscript letter indicating significant differences in each column (*p* < 0.05 by Duncan's test).

### 3.7. Proteolytic (prt) gene of PLAB-producing bioactive peptides

**Figure 4.**
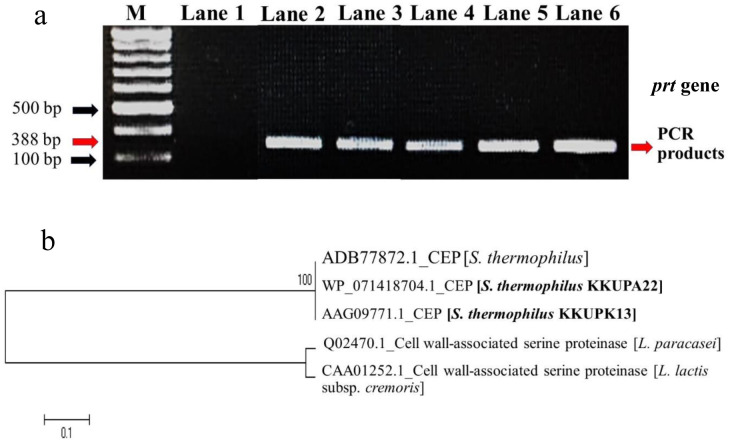
PCR products of specific amplified *prt* gene using *prt1-F* and *prt2-R* primers from each PLAB strains (a); phylogenetic tree of *S. thermophilus* strains KKUPA22 and KKUPK13 compared to reference strains, based on the *prt* gene sequences (b); M, DNA marker (VC 100 bp Plus DNA Ladder, Vivantis, Malaysia), Lane 1, *E. faecium* TISTR 2058 (negative control), Lane 2, *L. casei* TISTR1463, Lane 3, *L. Lactis* TISTR 1464; Lane 4, *S. thermophilus* TISTR 458; Lane 5, *S. thermophilus* KKUPA22; Lane 6, *S. thermophilus* KKUPK13. Bar indicates the interior-branch and bootstrap tests obtained by the neighbor-joining tree-building method.

It is of interest to examine whether the proteolytic activity *S. thermophilus* KKUPA22 and KKUPK13 is controlled by the *prt* gene. Two potent PLAB strains were designated the best PLABs that could produce highest proteolytic activity in the localization of both CB and EE activities on FFPB media. These PCR products were subjected to *prt* gene fragments of 388 base pairs with two specific primers *prt1-F* and *prt2-R* as shown in Figure 4a. The results of *prt* fragment sequences were similar to the region of *prtS* gene and established the relationship with *S. thermophillus* 4F44 (accession no. ADB77872.1) mentioned data showed the localization of the proteolytic enzyme on the active site of cell-envelope proteolytic type serine proteinase. When the prt gene sequences of KKUPA22 (accession number WP_071418704.1) and KKUPK13 (accession number AAG09771.1) were compared to those of bacteria in GenBank database, it was found that they were closely related to that of S. thermophilus 4F44 (accession number ADB77872.1) with 99.8 and 99.7% homology, respectively (Figure 4b). The results imply that S. thermophilus KKUPA22 and KKUPK13 would have similar proteolytic activity through the function of the prt genes as S. thermophilus 4F44 which hydrolyzed β-, αs-1-, and αs-2-caseins to generate biological bioactive peptides [5],[7]. Furthermore, in Figure 4b, S. thermophilus KKUPA22 and KKUPK13 were placed apart from Lactococcus and Lactobacillus species indicating the different mechanism of actions of their proteolytic enzymes. Similarly, numerous research has been conducted to demonstrate the close relationship between the operon proteolytic systems of *Lactococcus* and *Lactobacillus*, while *S. thermophilus* of proteolytic system exhibits additional traits that necessitate its own investigation [7],[11]–[13]. Resulting from *S. thermophilus* has been separated into three sections based on genetic investigations and *in vitro* and *in vivo* experiments; 1): a glutamine and methionine-deficient environment causes a serine proteinase attached to the cellular wall to become active. 2): the transportation of peptides and oligopeptides, which are both integrated in the Dpp system and the Ami system, respectively. It is important to note that the Ami system can transport peptides with up to 23 amino acids, whereas the Dpp system of *Lactococcus* or *Lactobacillus* can only transport chains with fewer than 13 amino acids. The last one, 3): peptide breakdown by intracellular peptidases, three of which are unique to *S. thermophilus* and are capable of either releasing aromatic amino acids or peptides containing aromatic amino acids [11],[60]–[62]. Also, several studies have been reported various CEPEs from LAB genomes in genera of *Lactobacillus* sp., including (*prt*B) from *bulgaricus* subsp. *bulgaricus* [60], (*prt*P) from *L. casei* or *L. paracasei* [61], (*prt*R) from *L. rhamnosus* [13], and (*prt*H) from *L. helveticus* [62]. These results are consistent with those of other studies and suggests that CEPEs start the hydrolysis of proteins by cleaving them into bioactive peptides of 4 to 30 amino acids, those peptides possess antioxidative and/or antimicrobial properties. Gene deletion tests have revealed that CEPEs are necessary for the strain to be developed in protein sources [63],[64]. Additionally, recent evidence suggested that the different transporters, including the oligopeptide permease (Opp), the ion-linked transporter (DtpT) for di- and tripeptides, and the ABC transporter (Dpp) for peptides comprising 2 to 9 amino acid residues, internalize the released extracellular peptides from CEPE cleavage. After that, numerous internal peptidases, including endopeptidases (PepO, PepF, PepE, and PepG), aminopeptidases (PepN, PepC, PepS, PepA, and PepL), tripeptidases (PepT), dipeptidases (PepD and PepV), and proline-specific peptidases, work together to degrade the internalized peptides into amino acids such as PepQ, PepI, PepR, PepX, and PepP [62],[63],[65],[66].

## 4. Conclusions

This study described the proteolytic activity of seven PLAB strains isolated from plaa-som samples. Both strains of *S. thermophilus* (KKUPA22 and KKUPK13) exhibited high extracellular and cell-bound proteolytic activity after incubation in fish flesh and skim milk protein media. The investigations showed that these PLAB strains having high probiotic, antioxidant and antimicrobial activity which might be from the generation of peptide-derived fish proteins. The proteolytic activity of the PLAB strains were confirmed to be associated with the *prt* gene controlling the operation of systematic CEPEs to degrade proteins to generate free amino acids or peptides. Therefore, this study would provide supportive information for further development of *S. thermophilus* KKUPA22 and KKUPK13 as starter cultures with a high proteolytic activity to catalyze protein hydrolysis and produce more bioactive peptide contents in functional food products.
